# 

**Published:** 1893-01

**Authors:** 


*Entered at the Post Office at Austin, Texas, as Second-Class Mail Matter.*

VOL. VIII. JANUARY, 1893. No. 7.

### DANIEL'S MEDICAL JOURNAL

SIT LUX

Subscription, $2.00 a Year, in Advance,—Single Copies, Twenty-five Cents.

F. E. DANIEL, M. D., Editor and Publisher, AUSTIN, TEXAS

EUGENE VON BOECKMANN, STEAM BOOK AND JOB PRINTER, AUSTIN, TEXAS.

